# Maternal Risk Factors for Fetal Alcohol Spectrum Disorders: Not As Simple As It Might Seem

**Published:** 2011

**Authors:** Philip A. May, J. Phillip Gossage

**Keywords:** Maternal alcohol consumption, prenatal alcohol exposure, maternal alcohol exposure, fetal alcohol spectrum disorders, risk factors, maternal risk factors, literature review

## Abstract

Gathering information about drinking during pregnancy is one of the most difficult aspects of studying fetal alcohol spectrum disorders (FASD). This information is critical to linking specific risk factors to any particular diagnosis within the FASD continuum. This article reviews highlights from the literature on maternal risk factors for FASD and illustrates that maternal risk is multidimensional, including factors related to quantity, frequency, and timing of alcohol exposure; maternal age; number of pregnancies; number of times the mother has given birth; the mother’s body size; nutrition; socioeconomic status; metabolism; religion; spirituality; depression; other drug use; and social relationships. More research is needed to more clearly define what type of individual behavioral, physical, and genetic factors are most likely to lead to having children with FASD.

Over the almost 40 years since fetal alcohol syndrome (FAS) was first described as a clinical diagnosis by Jones and Smith (Jones et al. 1973), several general maternal risk factors have been described in a number of studies using various approaches, including questionnaire-based surveys in prenatal clinics, surveillance using a variety of records, and population-based epidemiologic studies (May et al. 2009). One of the most difficult aspects of any research on fetal alcohol spectrum disorders (FASD) has been gathering accurate, honest, and detailed information on specific drinking patterns and actual or estimated blood alcohol concentration (BAC) levels and linking them to exact times of exposure in individual fetuses and children. Information on specific prenatal drinking behaviors that are the necessary causal factors for FASD has been elusive, and this has, in fact, limited the ability to determine the true prevalence of FASD more than any other factor (Eriksson 2007).

There are three major factors that must be addressed in the diagnosis of FASD in an individual: (1) physical growth, development, and structural defects (i.e., dysmorphology); (2) cognitive function and neurobehavior; and (3) maternal exposure and risk (Stratton et al. 1996). Of these three domains, detailed information on maternal drinking and cofactors of risk is most often missing for many cases. Without accurate and detailed maternal risk information, it is difficult to link specific, individual risk factors, or combinations thereof, to any particular diagnosis within the continuum of damage called FASD (Eriksson 2007). This article reviews highlights from the literature on maternal risk factors for FASD and illustrates that maternal risk is multidimensional, as there are a wide variety of variables that influence the development of a child with FASD. More research is needed to most clearly define what type of individual behavioral, physical, and genetic factors are most likely to lead to having a child with FASD.

When the diagnosis of fetal alcohol syndrome (FAS) was new in the medical literature in the mid-1970s, the link between alcohol use during pregnancy and FAS seemed simple. The literature was at first characterized by defining the unique traits of children with FAS, the most severe form of alcohol damage to the fetus (Clarren and Smith 1978; Jones and Smith 1973). Later, in 1981, the first Surgeon General’s warning on FAS simply stated: “The Surgeon General advises women who are pregnant (or considering pregnancy) not to drink alcoholic beverages and to be aware of the alcoholic content of foods and drugs” (U.S. Surgeon General 1981, p. 9). The simple truth reflected in the Surgeon General’s warning was that any woman who drank substantial amounts of alcohol during pregnancy could produce a child with FAS. But, to a great degree, no one was fully aware then of how much prenatal exposure to alcohol in any particular individual woman was necessary to cause the recognizable features of FAS that met the diagnostic criteria at the time. Some researchers believed that there might be a critical level of alcohol, a minimum “threshold” BAC that, once exceeded, would uniformly guarantee or produce FAS in children of the typical woman. However, as both early human and animal studies have shown, there is indeed a great deal of variation in the traits or features of FASD produced by individual mothers, different species of laboratory animals, and different animal strains within a species (Maier and West 2001; Thomas et al. 1996; West and Goodlett 1990). Because alcohol damage in humans ranges from mild to severe, examination of a variety of maternal behaviors and traits that might explain some or all of this variation is needed. Although some part of the differential vulnerability for the development of FASD likely is the result of genetic and epigenetic factors in the mother and/or fetus (Warren and Li 2005), evidence gathered to date suggests that the most substantial contributor to the variability in dysmorphology and other developmental deficits arises from differences in the extent of alcohol exposure, drinking pattern, and other maternal risk factors.

## Describing a Spectrum of Damage

At least two concepts emerged in research in response to the variable nature of the effects of prenatal alcohol exposure described in the literature from clinical and laboratory studies. The first was the concept that FAS is manifested in various levels of severity. The term fetal alcohol effects (FAE) (Aase et al. 1995) was first used to describe a number of traits similar to those found in FAS and, although less severe in their manifestation than in children with FAS, were linked to prenatal alcohol exposure and were evident in certain children born to mothers who were known to misuse alcohol. Traits of FAE were first recognized and the term coined in studies of laboratory animals. Some researchers questioned whether it was a viable term for use with humans in clinical settings and whether it was productive to label or provide a diagnostic term for these less severe manifestations of prenatal alcohol exposure in humans (Aase et al. 1995). Later, this continuum of effects was expanded to four different diagnoses by a committee of the Institute of Medicine (Stratton et al. 1996). The four diagnoses, from most dysmorphic to least dysmorphic, were designated as FAS, partial FAS (pFAS), alcohol-related birth defects (ARBDs), and alcohol-related neurodevelopmental disorder (ARND). The overarching term later coined to describe these four diagnoses was FASD (Warren et al. 2004).

Clinicians currently are more likely to diagnose children with FAS or pFAS than they are the less dysmorphic and growth-retarded cases such as ARND (Hoyme et al. 2005; Stratton et al. 1996). There are a number of reasons for this, but the following are two major factors: Severe dysmorphology and growth retardation represent the most recognizable traits of FASD, and the exact, unique neurobehavioral phenotype of FASD (especially ARND) has not yet been fully defined or developed. Furthermore, all population-based studies of FASD, to date, have used first-stage screening techniques based on dysmorphic features and physical growth retardation because dysmorphology currently is the most likely identifier of FASD.

The second concept that arose in an attempt to explain the variability of traits in alcohol-exposed children was the breakdown of maternal alcohol consumption by the quantity, frequency, and timing (QFT) of exposure. Defining alcohol consumption by specific traits of quantity, frequency, and variability (QFV) was first developed in epidemiologic studies of adult drinking (Mulford and Miller 1959, 1960). Using the concepts of QFV, these studies empirically described, in a manner that was particularly useful for researchers, the various drinking styles and patterns from survey data. This concept subsequently was adapted to the study of maternal drinking practices as they influence FASD. Briefly stated, the severity of damage to an individual child was, to a great degree, believed to be a function of the quantity (amount) of alcohol consumed by a mother during a pregnancy, the frequency (how often) that she consumed alcohol during that pregnancy, and the timing of the drinking during the gestation of the child (e.g., heavy drinking during the specific days when a particular anatomical feature of the fetus was developing) (May 1995).

Therefore, maternal risk for FASD initially was viewed within the two major frameworks outlined above. These held that if a woman drinks alcohol during a particular pregnancy, the child would be born affected to some degree, from mild to severe, depending on how much she drinks, how often, and the particular timing of the consumption during the pregnancy. Over the years, researchers (both basic scientists and epidemiologists) and clinicians have learned that it is not that simple. Other maternal traits and behaviors have been shown to play important roles in the variable nature of the features exhibited in alcohol-exposed offspring with and without FASD. The following sections will highlight first the QFT variables that are influential in maternal risk for FASD and then move on to describe other important maternal traits that have been linked to variation in severity of FASD traits in children across a number of studies.

## Binge Drinking and Severity of FASD: Quantity and Frequency Considered

The National Institute on Alcohol Abuse and Alcoholism (NIAAA) defines binge drinking among women as a pattern of drinking that brings BAC to 0.08 gram percent or above. For the typical adult woman, this pattern corresponds to consuming four or more drinks in about 2 hours (NIAAA 2004). Some studies of FASD have revised this definition to three or more drinks per occasion, as this level of drinking correlates highly with child dysmorphology and behavior (May et al. 2007, 2008). Binge drinking has been found to be the most damaging form of alcohol consumption on fetal development because it produces the highest BAC, and it is the peak BAC that affects the developing fetus most negatively (Abel 1998; Livy et al 2003; Maier and West 2001; Pierce and West 1986; West and Goodlett 1990).

Populations that have the highest rates of frequent binge drinking generally have been found to have the greatest number of babies born with FASD, particularly the most severe forms—FAS and pFAS (May et al. 1983, 2000, 2002, 2007; Urban 2008; Viljoen et al. 2005). Populations in which alcohol is consumed in a more moderate pattern, with lower amounts consumed over an extended period of time, generally will have fewer cases of FASD overall, more cases of pFAS than FAS, and more cases of ARND than FAS (May et al. 2006), but the ability of most clinicians to diagnose the majority of the less severe cases that are thought to exist still is limited.

By examining the ratio of only the two most severe forms of FASD to one another, one can gain an idea of the importance of binge drinking as a determinant of FASD severity. Table 1 shows the ratio of FAS to pFAS for several population-based studies. The populations listed in the top of the table have the highest proportion of heavy binge drinkers, and overall, the ratio of FAS cases to pFAS cases is higher in the communities where binge drinking is more prevalent. South Africa has a higher ratio of FAS cases to pFAS cases, primarily because it has the highest prevalence and most consistent pattern of weekly binge drinking, whereas Italy has the lowest occurrence of binge drinking. The normative pattern of drinking in Italy is moderate consumption of alcohol with meals, whereas heavy (binge) drinking on Friday and Saturday nights is the norm in the South African communities studied.

### Quantity of Alcohol Consumed

Longitudinal studies have documented lower overall cognitive and behavioral abilities among children born to women who report moderate or light drinking with infrequent binges (Jacobson and Jacobson 1994; Streissguth and LaDue 1985). In these studies, the mean IQ and other cognitive measures indicate that cohorts of children born to drinking mothers are deficient when compared with children of nondrinking mothers. The mothers’ alcohol use in these cohorts generally is not characterized as particularly heavy drinking or binge drinking; rather, the criteria are that the child was exposed to alcohol prenatally and the mean daily consumption exceeded 0.3 to 0.5 or more standard drinks per day as averaged across 7 days.

Therefore, quantity of alcohol consumed, particularly over a short period of time as in binge drinking, is the major factor in producing FASD. Alcohol is, as the name of the disorder indicates, the necessary condition. Moderate use of alcohol may not be a sufficient condition to produce FASD, although it can affect development, as noted above.

## Frequency of Alcohol Use

Frequency of use over 9 months of pregnancy also is a necessary condition to produce a child with FASD. Abel (1998) suggested that for FAS to occur, there must be frequent, heavy drinking over the course of the pregnancy and not just a few isolated episodes. Without regular occurrences of heavy drinking (e.g., weekly), then a diagnosable condition within the FASD spectrum is not likely to occur. In South Africa, study populations practice extremely regular binge drinking. Mothers of children with FAS and pFAS binge drink an average of 2 days every weekend, almost without fail, consuming an average of 6.6 standard drinks per evening (see table 2) on Friday and Saturday (May et al. 2000, 2007; Viljoen et al. 2005). In doing so, these particular women are producing BACs that are high enough and regular enough that their offspring have severe FASD (Khaole et al. 2004). In other words, given the composition of the population of this area, and the circumstances under which they live, the quantity and frequency of alcohol consumed are sufficient to produce very high rates of FAS and pFAS. The rate of FAS and pFAS combined in the most recent studies of the northern and western Cape provinces of South Africa have been 88 to 89 per 1,000 children (or 8.8 to 8.9 percent) in population-based studies (May et al. 2007; Urban et al. 2008).

The first population-based study of FAS (May 1991) provides another example of the necessity of both quantity and frequency occurring together for severe FASD to result. In the southwestern United States, seven communities of American Indians of three different cultural traditions were studied for FAS and what were at that time called FAE. The rates of FAS were highly variable between the different cultural groups, and the variation was based on the normative pattern of drinking, which affected frequency of drinking. Two of the communities were of tribal cultures that were more tolerant of heavy binge drinking on a sporadic basis than were the tribes of the other five communities. These two communities of Southwestern Plains tribal groups had the highest rates of FAS and FAE combined, because the sporadic binge drinking that was practiced among their women of childbearing age produced very high BACs. If the binge drinking did occur too frequently (e.g., daily or more than two times per week), it was not considered a serious breach of expectations within certain families and peer groups. In other words, the drinking was heavy but sporadic. Three of the other communities in this study were intolerant of heavy drinking among women in their tribal communities, especially of those women who had reached childbearing age. In these three communities, women who drank heavily were punished, jailed, or made to feel very uncomfortable. They often were ostracized (self-imposed in most cases) to off-reservation communities where the supply of alcohol was greater and the constraints on heavy consumption fewer, and therefore heavy drinking was more frequent. In these latter groups, the ratio of FAS to FAE was much higher (4.4 FAS cases to each case of FAE) because both quantity and frequency of drinking were high. In contrast, in the groups that were more tolerant of sporadic bingeing, quantities of alcohol consumed were high, but the frequency was not as great. This produced a rather equal number of FAS and FAE cases (1.4 FAS cases to each case of FAE) (May 1991).

## Survey and Questionnaire Information on Drinking During Pregnancy

Data on the extent of drinking during pregnancy in the United States and most other countries are believed to be inaccurate in that they may grossly underreport drinking in the prenatal period. The Centers for Disease Control and Prevention (CDC) has indicated that about 10.2 to 16.2 percent of pregnant women report drinking during the previous month, and 2 percent report binge drinking during that same time frame (CDC 2009). Yet studies of drinking prior to pregnancy recognition and retrospective studies have reported significantly higher levels, because recent studies have concluded that women who have reported their alcohol use after the fact, often long after the birth of a child and outside of prenatal clinics, are more truthful and accurate (Alvik 2006; Czarnicki 1990; Floyd et al. 1999; Hannigan et al. 2010; May et al. 2008). Fear of revealing prenatal drinking information prior to a child’s birth causes inaccurate reporting motivated by avoidance of shame and stigmatization. There have been a number of attempts to devise brief and somewhat indirect screening methods to determine whether there is alcohol exposure in a particular pregnancy (Chang 2001). These screening tools, although generally useful for clinical purposes, are inadequate for research purposes, which require data on differential levels and timing of exposure. Therefore, data on QFT obtained in prenatal clinics likely are very inaccurate (Hannigan et al. 2010), and prenatal clinics may provide the least accurate research information on drinking during the prenatal period. In fact, Hannigan and colleagues (2010) found that retrospective reports 14 years postpartum identified 10.8 times more women as at risk than in antenatal reports for the same women. Another excellent illustration of underreporting is a study from Sweden. Wurst and colleagues (2008) found that 8.7 percent of women in antenatal clinics interviewed with the AUDIT^1^ questionnaire reported drinking. The women also submitted urine and hair samples at the same time. When the samples were analyzed for fatty acid ethyl esters (FAEEs) and ethyl glucuronide (EtG), metabolites of alcohol that indicate recent consumption, the percentage of women who had actually consumed alcohol rose to 25.2 percent. Therefore, the methods and techniques for gathering accurate and specific research data on maternal risk have been inadequate in the past, especially in prenatal clinics. These must improve in the future with new, more effective questionnaire designs administered in appropriate settings and at times when the respondents will be most truthful and accurate (Alvik et al. 2006; Goransson et al. 2006; King 1994; Whaley and O’Connor 2003). Furthermore, better techniques of determining exposure by QFT, including biomarkers, are needed (Litten et al. 2010). Such improvements will not only improve research accuracy and understanding, but they also will aid clinicians in detecting alcohol use and abuse in prenatal clinics for intervention and prevention.

## Timing of Maternal Drinking and Effect on Children’s Physiology and Behavior

The timing of maternal drinking is critical as to which anatomical features are affected (Hoyme et al. 2005; Stratton et al. 1996; Sulik 2005; Sulik et al. 1981). Because of the sequential development of the fetus over an 8-to 9-month period, drinking during critical periods of gestation will produce various anatomical defects or brain-based cognitive or behavioral deficits, depending on the stage of development when a significant drinking episode occurs. For example, the key facial features that are commonly used to diagnose FAS and pFAS include short eye openings, thin border between the upper lip and facial skin, flat middle groove in the upper lip (i.e., philtrum), underdeveloped midface, wide distance between the right and left inner corners of the eyes (i.e., inner canthal distance), and droopy eyelid (i.e., ptosis). Each of these conditions develops during the sixth through the ninth week of gestation. If a woman’s drinking produces high BACs during this window of fetal gestation, then one or more of these features likely may be negatively affected and abnormal.

Timing also may be critical as to the particular cognitive and behavioral traits that are produced in a particular child. Even though the central nervous system, including the brain, is developing the entire 9 months of a normal pregnancy, particular critical regions (e.g., the hippocampus, regions of the frontal lobe, or corpus collosum) may have key windows in time when damage can result from a heavy binge or chronic drinking (Guerri et al. 2009; Mattson et al. 2001; Riley and McGee 2005). As studies continue to determine and define the specific nature of the behavioral characteristics of children with FASD, researchers may learn which regions of the brain are linked to particular deficits and behaviors and also when they are most at risk from the teratogenic effects of alcohol.

Therefore, the major necessary determinants of maternal risk factors for producing a child with diagnosable FASD are the quantity of alcohol consumed per occasion, the frequency with which drinking occurs, and the timing of these drinking episodes as they occur in relation to the specific gestational stages of the individual, developing fetus. Even though these conditions are necessary, and theoretically sufficient in the face of very high and frequent drinking episodes, they are not always sufficient as drinking is practiced by individual women and subgroups in society. That is, particular QFT levels of alcohol consumption that would produce FAS or pFAS in the offspring of a particular pregnancy of a particular mother may not do so in another pregnancy of another woman with different individual traits or cofactors of risk. Therefore, certain levels of alcohol exposure may not be sufficient to produce a child with FASD in the absence of certain other known cofactors of risk such as those detailed below.

## Maternal Characteristics That Modify Risk and Outcome: Age, Gravidity, and Parity

Given relatively similar reported QFT of drinking across pregnancies, it is possible for some children to be significantly more affected than others, even if they share the same mother. The sections below will examine the factors responsible for differential degrees of damage in the offspring of individual women (or individual pregnancies) who have reported drinking similar amounts of alcohol over similar time periods during pregnancy.

The first three maternal cofactors of risk that were identified by researchers are maternal age (chronological years), gravidity (number of previous pregnancies), and parity (number of previous births). Women who are higher on any of these three variables, on average, have been found to have children who are more severely affected than those borne to other women (Jacobson et al. 1996, 1998; May et al. 1983, 2005, 2006, 2007, 2008). In other words, the older the drinking pregnant woman is and the more pregnancies and children she has had, the greater the average likelihood that she will have a more severely affected child compared with other women drinking in a similar manner and at similar levels. Table 2 highlights these variables for studies from South Africa, Italy, and the Northern Plains of the United States. In each of these studies and populations, the mean gravidity and parity are higher in the maternal group bearing FASD children, and maternal age is higher in FASD mothers in all studies except one. Table 2 also shows that women who have children with FASD also have more miscarriages and stillbirth.

## Further Modifiers of Risk: Body Size, Nutrition, and Socioeconomic Status

In epidemiologic studies of FASD children in South Africa, Italy, and the United States, experience has shown that smaller women tend to be overrepresented in the FASD maternal group. As shown in table 2, the average height, weight, and BMI of the FASD mothers is lower than the control subjects in each country and sample. These differences are consistently and statistically significant in the larger samples such as the South African studies. In at least one cohort of the South Africa studies, head circumference of the mothers of FAS children was significantly smaller than the comparison group (May et al. 2005). This may indicate that some of the mothers of FASD children may have FAS or pFAS themselves.

As indicated in table 3, the average drinks per drinking day (DDD) measure is highest for the mothers of FAS children and lower for the other two groups: the mothers of pFAS children and the 24 percent of mothers of the randomly selected control children (children without FASD) who reported drinking during pregnancy. Interestingly, the average DDD measures of the mothers of some of the control children are equal to or higher than the average levels of the mothers of the pFAS children. Turning to the estimated average BAC levels for the three groups, however, the expected spectrum emerges as the BAC of the mothers of FAS children is highest, the pFAS mothers next highest, and the mothers of the control children the lowest. A major reason for this pattern likely is found in the maternal BMI. The mothers of the control children have the highest mean BMI, which reduces the BAC per drink and therefore, reduces alcohol exposure to the fetus. Body mass obviously and significantly moderates risk for FASD.

### Nutrition and FASD Risk

Nutrition studies of the average daily intake of foods among mothers in a small town and surrounding rural areas of South Africa have revealed that both mothers of children with severe FASD and mothers of control children have major nutritional deficiencies, placing them well below the recommended daily intake of both the United States and South Africa. This is undoubtedly one explanation for the very high rate of severe FASD in this region. Nevertheless, a comparison of the FASD mothers’ diet and that of control subjects indicates that the mothers of the FASD children have significantly lower intake of riboflavin, calcium, and DPA (one of the omega-3 fatty acids) than the mothers of non-FAS control subjects (May et al. 2004). Other nutrients, such as zinc and B vitamins, also may play a key role (Tamura et al. 2004). In fact, a recent study (Keen et al. 2010) indicates that a zinc deficiency was found in drinking mothers in both Russia and the Ukraine when compared with nondrinking mothers in the same antenatal clinics. Furthermore, a copper deficiency also was found in the Ukraine sample. The authors state that “select micronutrient deficiencies increase the risk for the occurrence of FASD in high risk populations. In theory these nutritional deficiencies can arise as a consequence of poor diets as well as a consequence of tissue injury-induced alterations in the metabolism of select nutrients” (Keen at al. 2010, p. 131). Therefore, undernutrition of a variety of nutrients is a risk factor for FASD for a variety of reasons over and above its effect on BMI. Although this is not a new concept to some basic scientists, it now is an increasing focus for researchers of FASD. Some researchers specifically are looking at using supplementation of particular nutrients (e.g., choline) both as a cofactor related to FASD damage and as a partial solution for reducing the damage caused by prenatal alcohol use (Thomas et al. 2004).

### Socioeconomic Status and FASD Risk

Although women of any socioeconomic status (SES) can bear children with FASD, the more severe forms of FAS and pFAS most frequently have been found in the lower SES categories in various countries. One classic study (Bignol et al. 1987) of the influence of SES in the United States found that the risk of bearing a child with FAS was 15.8 times higher for women of lower SES even with comparable drinking levels. Abel (1995) also identified lower SES as an important risk factor for FAS.

The SES of mothers of children with FASD is consistently lower than control subjects in epidemiologic studies as well. For example, all population-based studies of FASD in South Africa have indicated that the highest rates are found among women who live on the poorest rural farms where the living conditions are the worst, nutrition of the women is poorest, and weekend binge drinking is a regular practice. In most population-based studies, women with FASD children have lower levels of education and more frequently are unemployed or underemployed. Table 2 indicates clearly that this pattern holds in the South African, Italian, and U.S. studies represented, as maternal educational attainment is lower in all groups.

An overarching trait that may modify or enhance all of the above cofactors of risk is “weathering” (Holzman et al. 2009). Weathering is a concept put forth to explain the cumulative effect of poor living conditions, inadequate nutrition, and high levels of stress on childbearing. Research (Abel and Hannigan 1995; Bignol et al. 1987) has described the fact that women with lower SES on average have children characterized by lower birth weight and length, smaller heads, more malformations, and more attention deficit disorder, whether alcohol-exposed or not, and that diet and lower levels of nutrition, particularly antioxidants, are all enhanced risk factors in low-SES populations. Some studies in the United States have found that an early age of initiating regular drinking (May et al. 2005) may accelerate the weathering process by increasing the amount of time that alcohol can affect vital biophysiological processes such as the production of liver isoenzymes for alcohol metabolism, a change in the electrolyte balance in the digestive system, and longer-term exposure of the ovum to the teratogenic effects of alcohol.

## Metabolism and Known Genetic Influences

In the general clinical literature and in animal studies (Badger et al. 2005; Frezza et al. 1990; Shankar et al. 2006, 2007), it is known that alcohol metabolism varies from one individual woman to the next and that pregnancy affects alcohol and general metabolism in a variety of ways. This variance has both genetic and environmental influences. In a study in South Africa, researchers examined the effects of both metabolism and a known genetic polymorphism linked to alcohol metabolism among 10 women who had given birth to children with FAS, compared with 20 control women who had also consumed alcohol during pregnancy but borne unaffected children in the same birth cohort in the same town (Khaole et al. 2004). None of the women were pregnant at the time. They were allowed to drink beer or another beverage of choice at their own pace in a controlled situation in their own residence with the researchers present to monitor BAC via breathalyzer. The researchers found that the mothers of FAS children drank faster and produced high (peak) BACs of 0.20 more quickly. Furthermore, blood samples drawn from these women indicated that the mothers of FAS children were significantly less likely than the control women to have the protective genetic variants of the enzyme alcohol dehydrogenase^2^ (ADH) (i.e., ADH1B*2 and ADH1B*3). In other words, the mothers of the FAS children had the normal ADH variant of ADH1B*1 commonly found among the majority of human populations, those who can drink with fewer negative metabolism-related consequences (Khaole et al. 2004). Similar findings have been reported by others with the *ADH1B* pattern in other populations and studies (Jacobson et al. 2006; Vilijoen et al. 2001; Warren and Li 2005).

**Figure 1:**
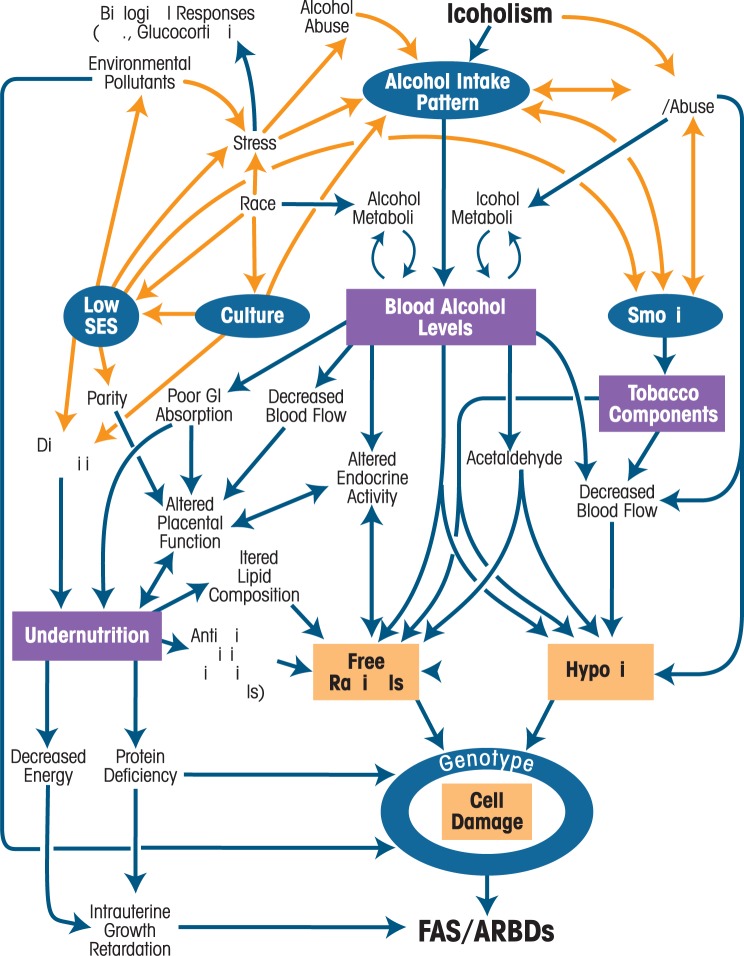
Schematic Summary of Permissive and Provocative Factors in FAS. Sociobehavioral permissive factors are shown in blue circles and biological provocative factors are shown in purple squares. Orange lines indicate associations among various environmental, demographic, and behavioral variables that can be bidirectional, whereas the blue lines indicate physiological pathways. SOURCE: Abel and Hannigan 1995. Reprinted with permission from the publisher.

Figure 1 shows a schematic summary (from Abel and Hannigan 1995) that illustrates the interaction of many maternal risk factors. In this figure, key variables of maternal risk, identified in both the human and animal literature, are depicted as influential, dynamic processes. Abel and Hannigan (1995) differentiate influential variables by classifying some as “permissive” and others as “provocative.” The permissive condition variables are those that “are predisposing behavioral, social, or environmental factors … that create the differential reaction to alcohol responsible for the occurrence/non-occurrence of FASD” (Abel 1988, p. 159). The provocative condition variables are those that are “related to physiological changes in the internal milieu … that increase vulnerability to alcohol’s toxic effects” (Abel 1988, p. 159). In this model, alcohol metabolism is considered in relationship to conditions and mechanisms that may permit and provoke the expression of traits of FASD. Key to this model is that undernutrition is associated with antioxidant deficiency, which permits the accumulation of free radicals. Free radicals increase the likelihood of cell damage and therefore make FASD traits more likely. Therefore, low SES, undernutrition, advanced maternal age, high parity, and overall weathering increase the risk for FASD trait expression in this scenario (Abel and Hannigan 1995).

## Religion, Spirituality, Depression, Other Drug Use, and Social Relations as Cofactors of Risk

In several studies in South Africa, two in Italy, and two in the United States (see table 2), women who reported less adherence to a major religion and less practice of prayer and regular church attendance were overrepresented in the maternal FAS group when compared with control subjects (May et al. 2005*a*, *b*, 2008; Viljoen et al. 2002). One of the Italian studies did prove to be a partial exception, as Italian women in the first study who gave birth to children with FASD were more likely to report a higher level of church attendance than control subjects (May et al. 2006) but were not necessarily higher on other measures of religiosity. Generally, women who are more likely to adhere to and practice a religious/spiritual tradition on a frequent basis (e.g., daily prayer) are less likely to drink and to drink to excesses that would cause FASD.

Depression has been reported to be more common among mothers of children with FASD (Flynn and Chermack 2008; Rubio et al. 2008; Trujillo Lewis 2008). Women who drink heavily and who have borne children with FASD are likely to have heavy drinking in their families of origin and procreation and also in their peer groups (Abel 1998*b*; May et al. 2005, 2008; Viljoen et al. 2002). The partners of women who bear FASD children are virtually always heavy drinkers or even very heavy drinkers of either a binge or chronic consumption style (see table 2).

Many studies indicate that mothers of FASD children in some countries use other drugs in addition to alcohol, as is evident in the two U.S. samples in table 2. South African and Italian women, however, are almost exclusively users of alcohol. Smoking also is much more common among mothers of FASD children (and drinkers in general) in all samples in table 2.

Domestic violence such as spousal abuse and poor domestic relations between parents of FASD children also are significantly higher in some studies (May et al. 2005, 2008). Households and families where FASD children are conceived, born, and raised tend to be less stable and more chaotic, which also may enhance the negative behavior traits that are often associated with children who have FASD.

## A Comprehensive Scheme for Organizing the Overall Risk for FASD

**Figure 2:**
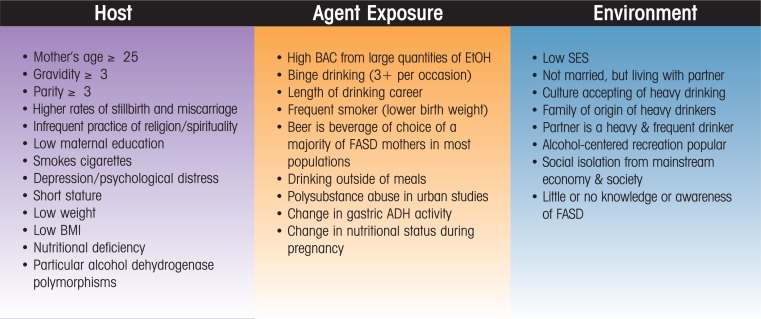
Commonly Recognized Maternal Risk Factors for FASD from the Literature: A Public Health Variable Summary.

As described above, many maternal factors affect FASD risk; figure 2 provides a useful scheme for organizing these variables. Using a standard public health classification (MacMahon and Pugh 1970) of associated and causal factors to organize the multiple, interdisciplinary variables that influence maternal risk for FASD in humans, a list emerges that may assist in clarifying our understanding of the multiple maternal influences on FASD. This schematic listing also may serve to guide further research, prevention, and intervention programming (May 1995). The three topical categories of variables are the host (the individual woman), the agent (alcohol as a teratogenic agent exposed to the fetus via the mother), and the environment (the social and natural setting of the pregnant woman’s life).

## Conclusions

Although research over the past four decades has identified many factors that contribute to the development of children with FASD, much work remains. Most importantly, detailed and accurate studies are needed to define the specific or average QFT of maternal alcohol consumption in women from specific populations that are found to produce children with each of the diagnoses within the continuum of FASD. For example, how many drinks per episode, how many episodes per week, and at which times during pregnancy does it take to produce a child with FAS, pFAS, or ARND among women in the general population of the United States (Hannigan et al. 2010; Robles et al. 1990)? Animal models provide many clues, but more accurate and specific studies of alcohol consumption in humans are greatly needed for advancements in research on maternal risk factors. Second, once this level of specificity is attained from improved maternal interviewing and other forms of data gathering from mothers, then other cofactors of risk can be controlled in statistical analyses, and the differential effects of variables such as gravidity, maternal age, body mass, nutrition, and other influences can be factored into the equation of risk and/or causation.

The major conclusion from this selective review, then, is that new and highly focused attention needs to be paid to gathering accurate and detailed data on maternal risk from mothers of FASD children with all levels of severity; from mothers who drank, but did not bear children with FASD; and also from those who do not drink. With specific and detailed data covering the variety of maternal risk factors over the entire course of pregnancies in representative, general populations, we can begin to definitively answer the complex questions of maternal risk for FASD. Improved methods of collecting maternal risk data are needed in order to make progress in this area of human study, especially since most people are reluctant to share such revealing and potentially stigmatizing information about themselves.

In 2005, the Surgeon General’s office updated the advisory on alcohol use and pregnancy. The new advisory reads: “We do not know what, if any, amount of alcohol is safe. But we do know that the risk of a baby being born with any of the fetal alcohol spectrum disorders increases with the amount of alcohol a pregnant woman drinks, as does the likely severity of the condition. And when a pregnant woman drinks alcohol, so does her baby. Therefore, it is in the child’s best interest for a pregnant woman to simply not drink alcohol” (U.S. Surgeon General 2005, p. 1)

Therefore, although much has been learned about individual maternal factors that both increase and decrease risk of FASD in individual offspring, the general warning appropriate for public health advice to the general population of women remains much the same: don’t drink alcohol when pregnant.

## Figures and Tables

**Table 1:** Cases of Fetal Alcohol Syndrome (FAS) and Partial FAS (pFAS) in Various Population Studies by Frequency, Percent, and Ratio

**Community Studies Organized From Top to Bottom by Proportion of Binge Drinking**	**FAS** *n* (%)	**pFAS** *n* (%)	**Ratio of FAS per pFAS**
South Africa I	40 (91)	4 (9)	10 to 1
South Africa II	37 (56)	29 (44)	1.3 to 1
South Africa III	55 (75)	18 (25)	3.1 to 1
Total South Africa*	**132 (72)**	**51 (28)**	**2.6 to 1**
Plains USA^**^	56 (45)	69 (55)	0.81 to 1
Western City, USA (1 & 2)^*^	6 (33)	12 (67)	0.5 to 1
Italy (1 &2)^*^	8 (18)	36 (82)	0.22 to 1

NOTES:

* All of these studies were school-based studies in which all consenting first-grade children were screened if their growth in height, weight, and head circumference was found to be below the 10th centile or they were picked randomly from the entire first-grade population as control subjects.

** Plains USA was an active-case ascertainment study in which children (birth to age 18 years) were recruited from seven communities to referral clinics for FASD and related developmental disabilities if they had physical features, behavior, or learning problems similar to those characteristics of FASD.

**Table 2:** Maternal Risk and Protective Factors From Studies of FASD: Selected Findings

**Variable**	**South Africa 1997, 1999, 2002 (*n* = 433) Mothers of:**		**Italy 2004, 2005 (*n* = 115) Mothers of:**		**Western City, USA 2007, 2008 (*n* = 72) Mothers of:**		**Northern Plains, USA 1997–2009 (*n* = 136) Mothers of:**	

	**FASD subjects**	**Control subjects**	**FASD subjects**	**Control subjects**	**FASD subjects**	**Control subjects**	**FASD subjects**	**Control subjects**
Age of delivery for index pregnancy [mean (SD)]	27.7 (6.5)	25.9 (6.1)**	31.1 (5.0)	29.3 (5.4)	26.8 (6.5)	28.2 (5.5)	26.6 (6.0)	24.1 (5.2)*
Rural residence during pregnancy (%)	51.4	26.6***	12.5	18.7*	0.0	0.0	75.8	93.1***
Educational attainment (years) [mean (SD)]	5.1 (3.2)	8.0 (3.0)***	Senior high school or higher (%)		High school or GED or higher (%)		High school or GED or higher (%)	
			37.5	71.1*	63.6	100.0***	54.8	92.0***
Involved in religion (%)	92.1	98.0**	85.7	93.4	90.9	91.5	86.7	93.3
Marital status (married) (%)	25.5	38.9***	100.0	92.4	54.5	83.3*	23.7	36.8*
**Childbearing**								
Gravidity [mean (SD)]	3.6 (1.6)	2.9 (1.3)***	3.4 (3.4)	2.4 (1.1)*	4.4 (2.1)	3.2 (1.6)*	5.2 (1.8)	3.7 (1.5)***
Miscarriages [mean (SD)]	0.3 (0.7)	0.2 (0.4)**	—	—	0.9 (1.4)	0.2 (0.7)*	0.6 (0.8)	0.3 (0.6)
Stillbirths [mean (SD)]	0.05 (0.2)	0.01 (0.1)*	—	—	0.0 (0.0)	0.0 (0.2)	0.1 (0.3)	0.1 (0.3)
Parity [mean (SD)]	3.3 (1.4)	2.7 (1.2)***	2.4 (2.7)	1.9 (0.6)	3.5 (1.9)	2.8 (1.2)	4.5 (1.9)	3.1 (1.4)***
**Women’s body profile**								
Height (cm) ([mean (SD)]	154.0 (5.9)	157.3 (7.0)***	156.3 (5.2)	162.8 (6.2)**	161.5 (7.6)	167.4 (7.6)*	163.6 (7.4)	163.3 (6.1)
Weight (kg) [mean (SD)]	58.0 (15.0)	68.2 (16.2)***	57.9 (8.3)	61.9 (8.8)	68.4 (12.9)	74.5 (18.6)	72.0 (17.6)	85.9 (18.8)***
Head circumference (cm) [mean (SD)]	54.4 (1.6)	54.8 (1.6)	—	—	—	—	55.2 (2.0)	56.0 (1.5)
BMI (kg/m^2^) [mean (SD)]	24.4 (5.9)	27.5 (6.5)***	23.0 (2.0)	23.3 (3.3)	26.4 (6.2)	26.7 (5.7)	26.9 (5.8)	32.4 (6.8)***
**Alcohol/drug use**								
Among drinkers, number of drinks consumed over 30 days by father of child during index pregnancy [mean (SD)]	110.9 (147.8)	83.6 (193.5)	—	—	78.2 (115.2)	33.4 (55.3)	276.1 (231.6)	142.2 (214.5)*
Age woman began drinking regularly [mean (SD)]	20.8 (4.3)	21.0 (4.4)	22.6 (7.8)	22.2 (6.8)	18.7 (3.1)	20.0 (5.6)	18.8 (4.5)	17.8 (3.2)
Among drinkers, number of drinks consumed by woman in week preceding interview [mean (SD)]	13.2 (12.1)	7.0 (6.6)***	16.6 (22.3)	2.1 (3.1)***	6.0 (0.0)	3.3 (2.9)	12.3 (11.9)	9.6 (6.0)
Among drinkers, number of drinking days by woman in week preceding interview [mean (SD)]	2.0 (1.0)	2.0 (1.3)	—	—	1.0 (0.0)	1.8 (1.0)	1.4 (0.9)	1.3 (0.5)
Woman used tobacco during index pregnancy (%)	77.7	34.8***	50.0	32.4	40.0	16.4	66.2	26.7***
Woman used other drugs during index pregnancy (%)	0.0	0.7	0.0	0.9	10.0	1.6	25.0	1.3***

NOTES:

* P < .05;

** P ≤ .01;

*** P ≤ .001; — Indicates that comparable data across populations do not exist in these individual studies, or maternal risk factor data have not yet been analyzed for these entire samples; SD = Standard deviation.

SOURCE: See May et al. 2006 for Italy; and Viljoen et al. 2002 and May et al. 2005 and 2008 for South Africa Waves I, II, and III. Specific details of the other two studies are not yet published independently.

**Table 3:** Average Drinks per Drinking Day, Estimated Peak BAC Levels,**** and Body Mass Index (BMI) Data from Interviews with South African Women (*n*= 175)

	**Drinking Mothers of Children with FAS**	**Drinking Mothers of Children with pFAS**	**Drinking Mothers of Children without FAS or pFAS†**
**1st trimester**			
D.D.D.^***^ (SD)	5.7 (3.8)	3.9 (1.4)	3.8 (3.4)^*^
BAC [mean (SD)]	0.197 (.17)	0.155 (.07)	0.122 (.11)
**2nd trimester**			
D.D.D. (SD)	5.7 (3.7)	3.2 (1.9)	3.7 (3.4)^*^
BAC (SD)	0.200 (.17)	0.124 (.09)	0.084^*^ (.09)
**3rd trimester**			
D.D.D. (SD)	5.5 (3.9)	2.7 (2.0)	3.7 (3.5)^*^
BAC (SD)	0.191 (.17)	0.102 (.12)	0.076 (.09)
Body Mass Index (SD)	22.5 (5.6)	23.5 (5.6)	27.4 (6.9)^**^

NOTES:

* p < .05.

** p < .001.

*** D.D.D. = avg. drinks per drinking day.

**** BAC estimated by the BACCuS technique (accounts for mother’s weight, quantity consumed, and duration of drinking).

† This group was selected from mothers of randomly selected non-FASD children in a community study of first-graders.

Specifically, this sample represents the 24 percent of mothers in this group who reported drinking during pregnancy.

SD = Standard deviation.

SOURCE: May et al. 2008.

## Footnotes

1 The AUDIT (Babor et al. 2001) is a 10-item screening questionnaire with three questions on the amount and frequency of drinking, three on alcohol dependence, and four on problems caused by alcohol.

2 Alcohol dehydrogenase (ADH) is one of the major enzymes involved in alcohol metabolism and converts alcohol to acetaldehyde, a toxic compound that can be damaging to the liver and other body organ systems. People with the ADH1B*2 and ADH1B*3 variants of the enzyme tend to have a more intense response to alcohol and a reduced risk for alcohol abuse and alcoholism.
